# Pandemics, economy and health in Asia-A scenario of post 2020

**DOI:** 10.1016/j.mex.2023.102347

**Published:** 2023-08-25

**Authors:** Devi Prasad Dash, Narayan Sethi

**Affiliations:** a School of Management and Entrepreneurship, Indian Institute of Technology, Jodhpur, Rajasthan, India; b Department of Humanities and Social Sciences, National Institute of Technology, Rourkela, Odisha, India

**Keywords:** COVID, Economic growth, Asia, Panel generalized least square models and IV-2SLS

## Abstract

Utilizing a daily data of 29 Asian Economies from June 2021 to June 2022, this study investigates the impacts of economic growth, health infrastructures and Government measures on COVID-19 cases. Our results demonstrate that GDP, Government intervention, testing and vaccination exert positive impacts on COVID-19 cases. We incorporate factors like weather to know how temperature impacts COVID-19 Cases. Our results demonstrate that magnitude of COVID-19 cases goes on upward fashion in winter days more. With reference to co-morbid conditions like diabetes, we notice that people with diabetes are more vulnerable to the infections, however due to the greater behavioral response, we obtain a negative association between co-morbid conditions and new COVID-19 cases. However, the intensity of COVID-19 cases is decimated with the improvement in health facilities and behavioral changes. Besides basic regression estimates, our instrumental variable estimates hold true in the line of regression results while underlying the relation with the COVID-19 cases. Interestingly, our results from alternate specification ensures that high human development with greater openness has resulted in more COVID-19 cases. Overall, our study belies the fact that vaccination and higher govt intervention can prevent COVID-19. Rather, a comprehensive policy is recommended on cross-country basis to overcome such challenge.

• The Study analyzes the relation among COVID-19, economic growth and health infrastructure on a daily basis from June 2021 to June 2022 for 29 Asian Economies
• Our empirical strategy involves regression followed by robustness tests of instrumental variable regression model.
• Results show that higher growth, human development, lesser vaccination and trivial govt intervention post 2020 have resulted in more COVID-19 cases.

## Graphical abstract

**Figure:**
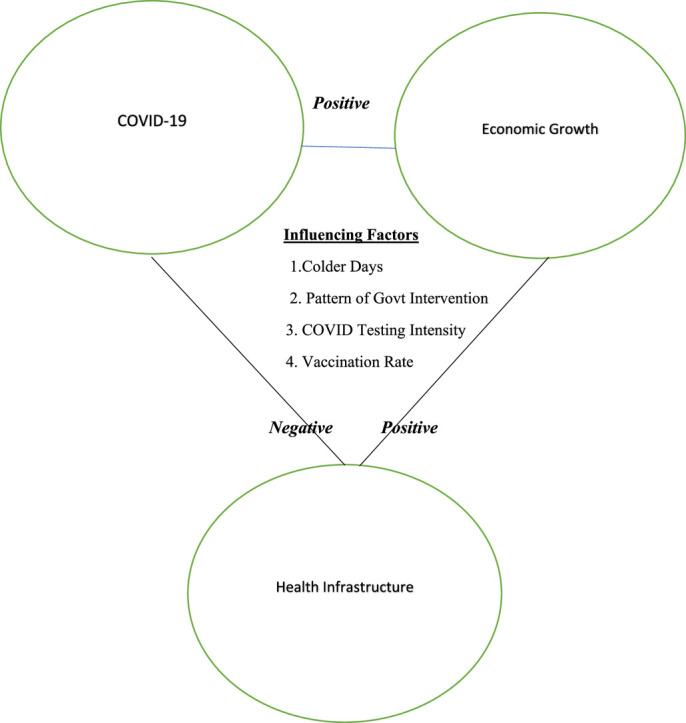

Specifications table

**Table:** 

Subject Area	Economics and Finance
More specific subject area	**VSI: COVID Research Robustness-Open**
Method name	Panel generalized least square models and IV-2SLS
Name and reference of original method	*Narayan, P. K., Iyke, B. N., & Sharma, S. S. (2021). New Measures of the COVID-19 Pandemic: A New Time-Series Dataset. Asian Economics Letters, 2(2)*. https://doi.org/10.46557/001c.23491
Resource availability	*NA*

*Method details

## Introduction

The COVID-19 outbreak has exerted devastating impact upon the economies globally. This study attempts to examine the economic impacts of COVID-19 for few Asian economies in this direction considering the cases of health infrastructures, co-morbid conditions, Government intervention and weather-related activities into the account. Economies especially in the South, South-Eastern, Eastern and neighboring parts have encountered economic severity and lockdowns with the challenges of mounting pressure on the health sectors post 2019. This study aims to flag some concerns post 2020 comprehensively in South and South-East Asia, considering the lethality and transmissibility of COVID-19 variants. Earlier literature in this direction fails to attain the comprehensive effects of COVID-19 post 2020 from the perspective of growth and health sector development [23], [24], [25], [26], [27]. Post 2020, economies have experience sudden drastic increase in COVID infection rates leading to the deceleration of economic growth, increased burden on the health sector and subsiding human development. This study has considered this context especially post 2020 scenario in order to see, how economic growth and health sector development stand amidst the Covid-19 challenges in the Asian economies.

COVID-19 has resulted in economic slowdowns, which in turn has led to firms’ shutdowns and employee lay-offs resulted in demand-supply mismatch across Asia [1,2,16,17]. Economic activity was significantly impacted in hotels, restaurants, retail, education, and the arts and entertainment [3], [4], [5]. Study by [6] unravels that the GDP growth rate decreases by 3–5 percent in a moderate scenario, costing the global GDP growth of around 2–2.5 percent for every extra month of closure. The COVID-19 epidemic contributed to the significant volatility that financial markets experienced during the global financial crisis more than ten years ago [7], [8], [9].

Past empirical work has significantly stressed upon the evidences of COVID-19 pandemic with various macroeconomic, financial and sectoral development for pre 2021 w.r.t scenarios. Most of the literature fail to address the economic and health sector scenarios aftermath 1st wave of COVID-19 prevalence especially in the Asian context. Our contribution in this direction is to show the evidence of linking COVID-19 infection with the human development, economic growth, govt initiatives and comorbid conditions of the people especially in the 2nd waves of infection prevalence in the developing blocs. We argue that economies especially in mid-2021 onwards have been highly infected with the virus’ infection upscale, which could be further underlined as the source of decimating economic growth, crumbling health infrastructure and high mortality in the region.

As of date literature has started focusing exclusively upon studying the impacts of COVID from the different sectoral perspectives. COVID has impacted economic and financial systems globally across economies to a various degree [18]. COVID-19 led uncertainty has triggered rapid fluctuation in oil price, exchange rate and stock market movements globally as well as in developing economy like India [19], [20]. Along with COVID induced fatalities, this pandemic led uncertainty has led to the declaration of economic activities globally in last 2 years [21], [22]. Given the developments in literature till date, this study seeks to investigate the impacts of both economic and non-economic factors upon COVID cases on a daily basis post 2020 scenario. We believe that such a study post 2020 will be able to capture an effective picture of COVID-19 handling and macroeconomic dynamic scenario in one of the highly COVID ravaged regions globally.

Building on the epidemiological SIR Model, we propose an economic model considering the role of human development, GDP and health infrastructures for the 29 Highly COVID-19 Ravaged Asian Economies post 2020 from June 1, 2021 to June 1, 2022.

## Literature review

In this section, we capture a brief analysis of previous literature pertaining to the economic effects of COVID-19 pandemics across globe and its repercussions. Considering several macroeconomic literatures on this COVID-19 infection transmission, it is well noticed that COVID led uncertainty has impacted global economy in varied manners. [28] have examined the impacts of COVID-19 on the African economy and noticed that COVID-19 induced lockdowns has impacted Africa's economic growth to a significant extent. [29] incorporate 7 possible implications of economic effects from COVID-19 in the European economies and found that declining reproductive rate and average GDP shortfall were of the greater concerns in the region. Intuitively, literature across economics and development studies have found various negative effects of COVID-19 across sectors ([30] on the banking sector; [31] on the water sector regulation; [32] on the marine sector management; [33] on the mental healthcare reforms; [34] on the childcare sector; [35] on the economic effects of livelihoods; [36] on the broadband sector). Mostly observations till date from prevalent body of literature suggest that existence of pandemics and its associated uncertainty have busted economic growth negatively in varied forms across the globe [25,37,38]. Considering the existing gamut of literature into the consideration, this study seeks to examine the impacts of COVID-19 upon the economy and health sector in particular post 2020 in order to evaluate the performance the sampled economies considered for our analysis.

## Data and methodology

We utilize the Our World in Data database in terms of retrieving daily data for 29 economies especially from South, South-East, East and Central Asian regions. This database mostly captures the information on a daily basis pertaining to some of the pressing issues globally. Data period considered in this manuscript are from 1st June 2021 to 21st June 2022 for all the 29 Asian Economies on a daily basis.^1^ This study undertakes one such pressing issue related to COVID-19 issues examining the association with the economic growth, weather, poverty, human development and other COVID-19 induced factors. The model uses a typical linear regression analysis. This study empirically utilizes COVID-19 cases as the outcome variable and subsequently incorporates economic growth, stringency, COVID-19 testing rate, weather dummy, handwashing facilities and hospital bed amenities. The rationale behind studying this analysis is to see, how emergence of new COVID-19 cases responds to changes in economic growth, testing intensity; weather related factors, Government interventions and basic healthcare facilities post 2020 scenario and second COVID-19 waves in particular. Most of the economies by June 2021 have experienced at least 1st waves and 2nd waves and few economies undergo the 2nd wave of COVID-19. The major motivation of the study is to see, how COVID-19 induced factors, economic growth and Government intervention go hand-in-hand even after 1.5 years of emergence of COVID-19 in Wuhan. We assume that emergence of new COVID-19 cases is endogenous. With reference to the information regarding variables concerned, we initially consider COVID-19 cases as the outcome variable in terms of numbers. Further, we consider GDP at constant price in terms of US$ daily as the main explanatory variable because of the fact that openness in terms of broadening economic activities influence the COVID-19 cases significantly in the region. In fact, the main reason behind coming up with a weather dummy is to see, whether summer or winter days influence cases more. We assign 1 for winter and non-winter days as zero. The 29 Asian Economies included in this analysis are Afghanistan, Bangladesh, Bhutan, Brunei, China, Hong Kong, Cambodia, India, Indonesia, Japan, Kyrgyzstan, Laos, Maldives, Malaysia, Myanmar, Nepal, Pakistan, Philippines, Singapore, South Korea, Sri Lanka, Russia, Mongolia, Iran, Taiwan, Tajikistan, Thailand, Uzbekistan and Vietnam. We provide a detailed description of each variable in Table 1 given below.

**Step-1:** We run a simple linear regression model considering both time and country effects followed by panel generalized least square models and PCSE model.
**Step-2:** Next, we incorporate the instrumental variable 2 SLS model to overcome the endogeneity arising out of regression analyses. This study proposes 4 different instruments to see, how GDP and various COVID-19 induced factors vary with new COVID-19 cases.

**Table 1:** Details regarding the variables of the study.

Variable	Description	Calculation	Source of Variable
COVC	Number of people catching COVID-19	In terms of numbers	Our World in Data
*GDP*	Final goods and services produced in an economy	Constant price in US $ on daily basis	Our World in Data
*HDI*	Human development considering education, longevity and percapita income	In terms of Index (0–100)	Our World in Data, originally taken from UNDP database
*WD*	Only considers winter days as the major proxy. It is a dummy variable	Winter days as 1 and other days as zero	Author's own estimation
*NT*	New testing of people in terms of identifying COVID-19 infection	In terms of numbers	Our World in Data
*FV*	Fully Vaccinated number of people	In terms of numbers	Our World in Data
*GIT*	It is a stringency index in terms of govt intervention, measuring the intensity of lockdown measures	In terms of Index ranging from 0 to 100	Our World in Data
*Diab*	People suffering from both type-1 and type-2 diabetes	In terms of number of people	Our World in Data
*HW*	Numbers of public hand washing facilities available in the hospitals, cities and other public areas	In terms of numbers of facilities	Our World in Data
*HB*	Numbers of hospital beds in total from all public and private hospitals from the standpoints of accessibility	In terms of numbers	Our World in Data

Source- Author's own compilation of data.

With reference to the empirical evaluation, we propose the following model with COVID-19 cases being the outcome variable, GDP being the major explanatory variable and weather, vaccination, stringency, health facilities as the control variables. The model is as follows.

(1) $$COVC_{it}=\alpha_{1}GDP_{it}+\alpha_{2}WD_{it}+\alpha_{3}NT_{it}+\alpha_{4}FV_{it}+\alpha_{5}GIT_{it}+\alpha_{6}Diab_{it}+\alpha_{7}HW_{it}+\alpha_{8}HB_{it}+\varepsilon_{it}$$

Where $COVC_{it}$ is the main outcome variable of the study in terms of numbers of COVID-19 cases. $GDP_{it}$ Indicates the gross domestic product at constant price for country *i* and year *t*. $\alpha_{1,2,--,8}$ are the parameters of the equation. $WD_{it}$ is the appropriate weather dummy in terms of differentiating winter and non-winter days.^2^ $NT_{it}$ and $FV_{it}$ represent the COVID-19 new tests and fully vaccinated individuals in terms of numbers respectively. Next, $GIT_{it}$ is denoted as the stringency index in terms of Government intervention having a score range from 0 to 100. $Diab_{it}$ reveals the number of people in a particular economy suffering from both type-1 and type-2 diabetes. We expect that diabetes prevalence and new COVID-19 cases exhibit positive association. $HW_{it}$ and $HB_{it}$ both represent handwash and hospital bed facilities in a country respectively and we expect it that both variables demonstrate inverse relation with the COVID-19 cases $\varepsilon_{it}$ is the idiosyncratic error term in the model. Further, we consider human development in place of GDP. The motivation behind undertaking such step is to see, how COVID-19 cases are being impacted with the rise in human development. We normally find that human development is considered as the resultant factor of improvement in GDP growth. Our empirical specification is as follows,

(2) $$COVC_{it}=\beta_{1}HDI_{it}+\beta_{2}WD_{it}+\beta_{3}NT_{it}+\beta_{4}FV_{it}+\beta_{5}GIT_{it}+\beta_{6}Diab_{it}+\beta_{7}HW_{it}+\beta_{8}HB_{it}+\varepsilon_{it}$$

In above Eq. (2), HDI is being considered as the main explanatory variable in place of GDP in the earlier equation. $\beta_{1,2,--,8}$ are the parameters for the Eq. (2).

## Empirical results

At the outset, in our empirical strategy, we report the descriptive statistics for each of the variable used in our model. The descriptive statistics are reported in Table 2 as follows.

**Table 2:** Descriptive Statistics.

Variables	COVC	GDP	HDI	WD	NT	FV	GIT	Diab	HW	HB
*Mean*	2.658	3.888	0.703	0.172	2.170	4.185	1.608	0.906	1.069	0.470
*S. D*	1.350	0.854	0.174	0.377	2.338	3.530	0.434	0.211	0.903	0.314
*Max*	5.793	4.932	0.949	1	7.554	9.100	1.979	1.248	1.985	1.147
*Min*	0	0	0	0	0	0	0	0	0	0

*Notes:* All variables are converted into natural logarithm. WD refers to the weather dummy. Author's own compilation.

The Table 3 reporting these estimates state that improvements in GDP and human development have led to a rise in COVID-19 cases. It further shows that when economic activities, business movement and connectedness improve, then it has led to the rise in COVID-19 cases. Our finding of positive association between HDI and COVID-19 infection is in the line of few studies like [14,16]; Further, evidences from weather pattern depicts that infection rate remains high in the time of cold months, which is consistent in the line of several other studies [10,11]. Other factors like testing and vaccination still exhibit positive association with the COVID-19 cases over the months of 2021–22 (See Table 3). It suggests that the vaccination rate in the region remains unequal and evidences also state that fully vaccinated people again have been prone to mild COVID-19 attack, which is consistent with [15]. In case of exploring relation between intervention and COVID-19 cases, we find that Government response in our case has been least effective in controlling the cases. Our empirical results state that there has been a significant increase in COVID-19 infection rates despite higher Government intervention. Our finding is also in the line with [12,13,16]. Positive impacts indicate that rise in GDP, govt intervention, testing and vaccination results in more COVID-19 cases. Empirical results signify that rise in economic growth leads to more openness, which in turn results in more interaction of people, leading to more COVID-19 infection within and across economies. More testing results in identification of more covid cases. While considering vaccination, we find that people even if being vaccinated, started being impacted in COVID-19 when there is a greater degree of openness, interaction at different spheres (See Table 3). This still somewhere reflects the efficacy of vaccination impacts as a whole during 2021–22.

**Table 3:** Regression Results.

COVC	I	II	III	IV	V	VI
*GDP*	0.449* (0.023)	0.461* (0.022)	0.462* (0.051)			
*HDI*				1.554* (0.111)	1.618* (0.113)	1.973* (0.242)
*WD*	0.301* (0.026)	0.337* (0.026)	0.142* (0.053)	0.286* (0.027)	0.318* (0.023)	0.136* (0.053)
*NT*	0.258* (0.004)	0.278* (0.003)	0.084* (0.006)	0.253* (0.004)	0.271* (0.003)	0.083* (0.006)
*FV*	0.056* (0.003)	0.044* (0.002)	0.008* (0.001)	0.055* (0.003)	0.043* (0.002)	0.008* (0.001)
*GIT*	0.578* (0.023)	0.559* (0.018)	0.442* (0.049)	0.599* (0.024)	0.567* (0.019)	0.456* (0.049)
*Diab*	−0.772* (0.084)	−0.536* (0.073)	–0.977* (0.173)	−0.249* (0.077)	0.055 (0.066)	−0.638* (0.167)
*HW*	−0.065* (0.012)	−0.051* (0.012)	−0.102* (0.031)	−0.030* (0.012)	0.010 (0.011)	−0.058** (0.032)
*HB*	0.382* (0.038)	0.437* (0.041)	0.245* (0.074)	0.406* (0.041)	0.513* (0.046)	0.194* (0.078)
*Constant*	−0.275* (0.065)	−0.485* (0.069)	0.785* (0.162)	−0.164* (0.066)	−0.356* (0.069)	0.852* (0.160)
*Method*	Simple Regression	Panel GLS	PCSE	Simple Regression	Panel GLS	PCSE
*No of obs*	11,194	11,194	11,194	11,194	11,194	11,194
*R square*	0.389		0.159	0.378		0.157

*Notes:* Standard errors are given in parentheses. (∗), (∗∗) and (∗∗∗) denote significance at 1%, 5% and 10% respectively.

Author's own compilation. There is no reporting of R square in panel GLS.

We now provide a comparison of our region-specific results in order to draw more robust analysis by setting up the basic linear regression model. Based on the economic diversity, connectedness, growth and openness, the region has been divided into 3 different blocs- South Asia, South-East Asia and East-Central Asia (See Table 4). The empirical investigations are being carried out considering GDP and HDI differently in order to see, how COVID-19 infection rates are being impacted through various factors. We argue that improvement in economic development has helped contain the growth of infection rates successfully in S-E Asia, while the reverse becomes true for South and East Central Asia because of higher openness and connectedness. The same gets reflected in case of human development for South and East-Central Asia. We further apply the effects of winter and colder days to check, how weather impacts the COVID infection rate. Our empirical investigations show that a positive and significant results emerge between infection rate and colder days. We further conduct a detailed analysis with reference to the vaccination drives and govt stringency in terms of inhibiting the virus spreading. Our empirical results report that despite the stricter implementation of govt initiated lockdowns, containments and vaccination drives, virus spreading remains robust across these regions. The plausible explanations could be due to the higher population density, lack of awareness drives and unequal healthcare accessibility. Further, our results in relation to the co-morbid conditions define that people with diabetic and pre-diabetic conditions are highly vulnerable to catch COVID-19 infection. Next, our analysis in terms of examining the cases of health infrastructure likes availability of hospital beds reveal that the situations in S-E and East-Central Asia remain grim in terms of admitting COVID positive patients in the hospitals. Notably, the situation remains better in South Asia in terms of the availability of hospital beds concerned. For our baseline estimates, it is evident from the comparative perspectives that availability of health infrastructure and basic knowledge of cleanliness drives and facilities across people remain different across regions.

**Table 4:** Comparative Analysis of the Regions.

COVC	I	II	III	IV	V	VI
*GDP*	0.449* (0.058)		−0.505* (0.077)		0.820* (0.045)	
*HDI*		0.717* (0.273)		0.411 (0.302)		2.964* (0.234)
*WD*	0.062** (0.036)	0.060** (0.036)	0.563* (0.069)	0.633* (0.069)	0.269* (0.038)	0.297* (0.038)
*NT*	0.201* (0.006)	0.204* (0.007)	0.206* (0.007)	0.206* (0.009)	0.035* (0.011)	0.035* (0.012)
*FV*	0.037* (0.004)	0.038* (0.004)	0.048* (0.004)	0.045* (0.004)	0.017* (0.006)	0.025* (0.006)
*GIT*	1.154* (0.033)	1.091* (0.036)	0.424* (0.053)	0.447* (0.053)	0.944* (0.055)	0.877* (0.056)
*Diab*	3.318* (0.421)	4.042* (0.412)	3.300* (0.198)	2.900* (0.214)	−4.363* (0.225)	−3.361* (0.264)
*HW*	−1.101* (0.299)	−1.042* (0.035)	0.657* (0.043)	0.805* (0.038)	−0.467* (0.023)	−0.509* (0.023)
*HB*	−4.689* (0.146)	−4.616* (0.149)	2.575* (0.172)	1.938* (0.181)	1.794* (0.059)	1.943* (0.052)
*Constant*	−4.988* (0.437)	−4.386* (0.385)	−1.429* (0.363)	−3.476* (0.258)	0.222* (0.106)	0.329* (0.109)
*Region*	South Asia	South Asia	S-E Asia	S-E Asia	East and Central Asia	East and Central Asia
*R^2^*	0.590	0.584	0.482	0.476	0.545	0.527

*Notes:* Standard errors are given in parentheses. (∗), (∗∗) and (∗∗∗) denote significance at 1%, 5% and 10% respectively.

Author's own compilation.

As seen from Table 5, we find that rise in lockdown time period has actually resulted in more COVID-19 cases. It is quite imminent from the fact that lockdown strategies have not been properly adhered to the designated strategies and flouted by citizens in several instances. Simultaneously, we also obtain positive and significant association between economic growth and rising of new cases, which itself shows that more opening up of economies have led to the rising in infection rates again. Even if growth is instrumented w.r.t vaccination drives and higher testing, still then the number of cases is found to remain high in 2021–22.

**Table 5:** Robustness Checks-Instrumental Variable 2 SLS (IV-2SLS) Regression with GDP being the explanatory variable.

COVC	I	II	III	IV
*GDP*	0.551* (0.099)	0.861* (0.114)	0.156* (0.056)	−1.016* (0.094)
*WD*	0.547* (0.130)	0.209* (0.033)	0.189* (0.042)	
*NT*			0.270* (0.060)	0.112* (0.029)
*FV*			0.174* (0.014)	0.259* (0.039)
*GIT*	1.648* (0.728)	0.816* (0.028)	0.890* (0.045)	
*Diab*	−1.122* (0.372)	−2.242* (0.355)	−0.163 (0.157)	1.964* (0.342)
*HW*	−0.861* (0.082)	−0.064* (0.014)		−0.081* (0.034)
*HB*	−0.606* (0.182)	−0.169** (0.101)		1.365** (0.829)
*Constant*	−1.742* (0.244)	0.140 (0.117)	0.592* (0.149)	1.088** (0.764)
*Instruments*	GIT = NT FV	GDP = NT FV	NT = HW HB	GDP = WD GIT
*Durbin Chi-square P value*	326.65* (0.000)	12.425* (0.000)	159.93* (0.000)	270.20* (0.000)
*Wu-Hausman F test P value*	461.43* (0.000)	12.430* (0.000)	162.13* (0.000)	276.69* (0.000)
*First stage F test*	121.39*	314.25*	70.629*	19.93*

*Notes:* Standard errors are given in parentheses. (∗), (∗∗) and (∗∗∗) denote significance at 1%, 5% and 10% respectively.

Author's own compilation.

Here in Table 6, we replace GDP with HDI being the major explanatory variable. We find unexpectedly a positive association between HDI and COVID-19 cases. However, it shows negative relation in case HDI being instrumented for weather and Government intervention (See Table 5). It primarily shows that weather and Government intervention help dampen the COVID-19 incidents. Like earlier models, our empirical results depict positive association between winter days and infection rates. Mostly, COVID-19 spreading increases in colder days. Results from full vaccination are not that encouraging, stating that there exists still positive correlation with new cases. In certain cases, our results demonstrate that improvements in hospital bed facilities, handwashing facilities and government intervention in turn results in more COVID-19 spreading (See Table 6).

**Table 6:** IV-2SLS Model in presence of HDI being the explanatory variable.

COVC	I	II	III	IV
*HDI*	4.987* (0.451)	1.334* (0.339)	0.357 (0.320)	−1.716* (0.575)
*WD*	1.779* (0.637)	0.266* (0.035)	0.179* (0.045)	
*NT*	−0.453* (0.118)		−0.299* (0.083)	0.121* (0.020)
*FV*			0.181* (0.020)	0.356* (0.033)
*GIT*			0.907* (0.054)	
*Diab*	−2.165* (0.321)	−2.146* (0.246)	0.103 (0.099)	1.919* (0.956)
*HW*	0.900* (0.077)	0.132* (0.019)		−0.731* (0.079)
*HB*	−1.170* (0.187)	−1.260* (0.104)		1.256* (0.130)
*Constant*	−1.555* (0.111)	−0.534* (0.108)	0.713* (0.171)	4.317* (0.408)
*Instruments*	GIT = NT FV	HDI = NT FV	NT = HW HB	HDI = WD GIT
*Durbin Chi-square P value*	305.3*(0.000)	261.69*(0.000)	96.26* (0.000)	717.36* (0.000)
*Wu-Hausman F test P value*	419.4* (0.000)	267.76* (0.000)	97.03* (0.000)	765.93* (0.000)

*Notes:* Standard errors are given in parentheses. (∗), (∗∗) and (∗∗∗) denote significance at 1%, 5% and 10% respectively.

Author's own compilation.

## Conclusion

This study investigated the nexus among COVID-19 cases, GDP, HDI, weather and various COVID-19 induced factors for 29 Asian economies post 2020. The results from full sample show that higher GDP and longer winter days accelerate the COVID-19 infection rate across the region. Full vaccination in the region as a whole exerts mild impact in controlling COVID-19. It is confirmed that triple strategies like high Government intervention, higher vaccination rate and improved testing intensity help decimate COVID-19 wave to a greater extent. Moreover, findings from our study confirm that in the region, joint COVID-19 strategy is still lacking, as evident from vaccination scale. Interestingly, we notice that hospital bed facilities overall in the region have been increased significantly, which itself indicates that COVID-19 has pushed the economies in the region to invest heavily in healthcare sectors. We also find that changing behavioral perspectives in terms of at least hand washing frequently has led to the controlling of COVID-19 significantly. In light of these findings, this study recommends that joint strategies in controlling COVID-19, equal vaccination rate, private participation in healthcare sector, higher testing rate and stimulating more public awareness ensure the smooth functioning of economies in long run. The study further suggests that improving GDP and opening up of economies should not be compromised at the cost of adopting a lackadaisical approach towards COVID-19 handling. Apart from the length of lockdown, several critical factors like health infrastructure, co-morbid conditions and macroeconomic characteristics have been identified in terms of analyzing the post 2020 Covid-19 scenario in these Asian economies. Considering such a wide diversity across economies, the study feels that a pan Asian health organization needs to be established to address the health inequality and backwardness in these economies. Second, health and economic opportunities for the socially disadvantaged, vulnerable groups along with the special focus on children and women need to be enhanced in these economies with a higher stake of private sector participation. Third, initiatives like social safety schemes, low cost mobile money transfer and innovative delivery approaches of medicine and health care facilities need to be deployed at the massive scale especially in the rural, semi-urban areas of the region.

## Declaration of Competing Interest

The authors declare that they have no known competing financial interests or personal relationships that could have appeared to influence the work reported in this paper.

## Data Availability

Data will be made available on request.

## Footnotes

1 Please find the data in daily frequency format from this link only- https://github.com/owid/covid-19-data/tree/master/public/data. (From Our World in Data database). Daily frequency data of all variables considered here including daily percapita GDP data except weather dummy are retrieved from this above link specified here.

2 While fixing the weather dummy, we consider the winter time for each economy. For winter days, we assign value as 1, rest other days are as zero. Even if there are few South Asian economies, where there is absolutely no winter. For such economies, we consider all the days of the year as zero. The scientific research shows that covid-19 virus tends to spread faster particularly in winter time.
